# Scoliosis Orthopedic Surgery Combined With Nusinersen Intrathecal Injection Significantly Improved the Outcome of Spinal Muscular Atrophy Patient: A Case Report

**DOI:** 10.3389/fneur.2022.869230

**Published:** 2022-04-25

**Authors:** Beiyu Xu, Cuijie Wei, Xiao Hu, Wenzhu Li, Zhen Huang, Chengli Que, Jianxing Qiu, Chunde Li, Hui Xiong

**Affiliations:** ^1^Department of Orthopedic/Spine Surgery, Peking University First Hospital, Beijing, China; ^2^Department of Pediatrics, Peking University First Hospital, Beijing, China; ^3^Department of Anesthesiology, Peking University First Hospital, Beijing, China; ^4^Department of Rehabilitation Medicine, Peking University First Hospital, Beijing, China; ^5^Department of Respiratory and Critical Care Medicine, Peking University First Hospital, Beijing, China; ^6^Department of Radiology, Peking University First Hospital, Beijing, China

**Keywords:** SMA, scoliosis orthopedic surgery, Nusinersen intrathecal injection, real-time ultrasound guidance, CT guidance, case report

## Abstract

**Background:**

Spinal muscular atrophy (SMA) is an autosomal recessive disorder caused by pathogenic variation of the survival motor neuron (SMN) 1 gene. Symptoms of SMA include progressive limb muscle weakness, atrophy, and severe scoliosis. Nusinersen is an antisense oligonucleotide that can enhance the production of the SMN protein. Here, we report a case with scoliosis who received orthopedic surgery combined with Nusinersen intrathecal injections.

**Case Presentation:**

Scoliosis orthopedic surgery followed by Nusinersen intrathecal injections was given to a 16-year-old girl who had thoracic and lumbar scoliosis and type 3 SMA. Surgery was performed for T3-S2 posterolateral fusion (PLF), with a channel left on the vertebral laminae of L3-L4. The balance of the spine and pelvis was significantly improved and the height increased by 9 cm. Lumbar puncture was conducted with local anesthesia under ultrasound and CT guidance through the laminae channel and Nusinersen was successfully injected. Comparing the two approaches, real-time ultrasound guidance for intrathecal Nusinersen injections after spinal surgery is preferred, however, CT guidance is an alternative if the initial puncture procedure is difficult. After the aforementioned multidisciplinary treatment, a good outcome was achieved, as demonstrated by a 2-point increase in RULM and MFM32 scores 2 months later.

**Conclusion:**

Scoliosis orthopedic surgery combined with Nusinersen intrathecal injection is an effective treatment for SMA patients with scoliosis.

## Introduction

Spinal muscular atrophy (SMA) is an autosomal recessive disorder that leads to the degeneration and even loss of the motor neurons in the spinal cord. Usually, it is caused by the deletion of exon 7 of the SMN1 gene. It has been noted that its incidence is ~10 in 100,000 at birth and later prevalence of individuals with classic forms of the disease is ~1–2 per 100,000 persons (1). According to the age of onset and maximum exercise capacity, there are three different types of SMA, namely, type 1, type 2, and type 3, in children, and type 4 in adulthood. Among those, type 1 SMA is the most severe and also the most common fatal neuromuscular disorder in infants (2). SMN2 gene undergoes alternative splicing and produces a truncated mRNA isoform missing exon seven. A C-to-T nucleotide transition at position six of exon seven of the SMN2 gene is responsible for the alternative splicing. The resulted truncated SMN protein is non-functional and thought to be degraded rapidly. About 10% of SMN2 pre-mRNA is properly spliced and subsequently translated into full-length SMN protein. Therefore, the quantity of the functional SMN protein produced and the clinical severity of SMA is dependent on the number of the SMN2 gene copies retained in the SMA patients.

Nusinersen is an anti-sense oligonucleotide, which was designed to modify the splicing behavior of the SMN2 gene and thus increase the inclusion of exon7 and the expression of the full-length SMN protein (3, 4). It was approved by the US Food and Drug Administration (FDA) in late December 2016, the European Medicines Agency in June 2017 (5), and the Chinese national medical product administration (NMPA) in 2019. Due to its inability to pass through the blood–brain barrier, Nusinersen can only be administered intrathecally through lumbar puncture (6).

Respiratory dysfunction is the most common complication and primary cause of death in SMA. Patients may have difficulty in clearing lower respiratory secretions due to cough weakness, and some with severe scoliosis may develop restrictive ventilatory dysfunction due to their severe chest wall deformity. Scoliosis and hip subluxation or dislocation are the two most common orthopedic problems encountered by patients with SMA (3). The collapsing long C-shaped scoliosis (7), typically seen in children with SMA, is caused by the weakness of the trunk muscles and their inability to keep the spine in the upright position (4). Such curvature may involve a large segment of the spine with its apex often located in the thoraco-lumbar junction, extending into the pelvis and leading to the development of a significant imbalance between trunk and pelvis (8). The age of onset, severity, and progression of scoliosis are related to the type of SMA and the extent of muscle weakness (9). The more severely the child is affected, the higher the probability of developing scoliosis at an earlier age and also the greater anticipated curve progression (1). Spinal fusion is recommended in the presence of documented scoliosis progression and curves between 40° and 60° in children, ideally at 10 years of age or older (10), especially, if there is a recorded deterioration in their motor function or pulmonary capacities. However, intrathecal injection treatment is very difficult for the patients who have received orthopedic surgery because of the lack of anatomical landmarks on the vertebral laminae and deficient flexion of the lumbar spine after scoliosis orthopedic surgery and there were few experiences in China up to date.

Here, we report an SMA case with scoliosis who underwent orthopedic surgery followed by Nusinersen intrathecal injections, and technical essentials of the procedure. It perfectly reflects the multidisciplinary cooperation.

## Case Report

### Clinical Data

The case is a 16-year-old girl who achieved independent walking at one year old. She was found to have progressive muscle weakness with waddled gait and difficulty walking up and down stairs and squatting at 3 years old. She developed tremor in both the hands at the age of 5 years. At the age of 12, the patient was unable to squat or walk up and down stairs. She lost her ability to walk at 13 years old and ability to stand at 15 years old. Currently, she is wheelchair bound. The muscle strength of the patient's upper limbs is relatively preserved compared to her lower limbs and she can write in a prone position, but cannot lift heavy objects. Her intelligence is normal. There is no consanguinity in the parents and no history of any neurological disorders, genetic conditions, or early deaths in either parents' family.

On examination, the patient had atrophic muscles in her proximal lower limbs and relatively pseudohypertrophy of the gastrocnemius. Her upper extremity strength was Grade 4 and lower extremity strength was Grades 2-3, based on Medical Research Council (MRC) scale. She was observed exhibiting tongue fasciculations. She has tongue fasciculation and atrophy, areflexia, joint contractures, ankylosis of both feet, and severe scoliosis.

Her EMG showed neurogenic injury with abnormal insertional activity and reduced recruitment pattern. The muscle MRI showed fatty infiltration, amyotrophy, and edema.

### Diagnostic Tests

Her genetic testing showed homozygous deletion (exon7 and exon8) of the SMN1 gene, and there were 3 copies of the SMN2 gene. Then, the systemic functions were assessed in detail after diagnosis.

### Orthopedic Complications

Scoliosis and pelvis obliquity developed after the patient lost her independent walking. She was diagnosed to have long C-shaped thoracolumbar scoliosis with cobb angel of 63° at present. Inclination of the pelvis was 9.5°. Because of the inclination of pelvis and scoliosis, she needs to use both hands to maintain balance while sitting, therefore, making it impossible for her to study or work with her upper limbs.

### Respiratory Involvement

Pulmonary function test showed a decrease vital capacity and an increased residual volume. Her diffusing lung capacity for carbon monoxide (DLCO) was 79.45% predicted. The patient's lung function damage was presented as restrictive ventilatory defect.

## Scoliosis Orthopedic Surgery

Rapid progression of scoliosis and decompensation of the trunk can lead to cardiorespiratory functional impairment in an unexpectedly short period of time. In order to free up the upper limbs for daily activities and to prevent cardiorespiratory functional deterioration, spinal fusion is recommended for her. In consideration of the pelvis obliquity and the flexibility of scoliosis, an isolated posterior spinal arthrodesis should be sufficient to achieve satisfactory correction of her deformity, without the need for additional anterior surgery or preoperative spinal traction. Scoliosis orthopedic surgery with sequential Nusinersen intrathecal injection was decided with fully consent from both the patient and her parents. She received the T3-S2 PLF surgery with 30 pedicle screws implanted in September 2021.

**Figure 1:**
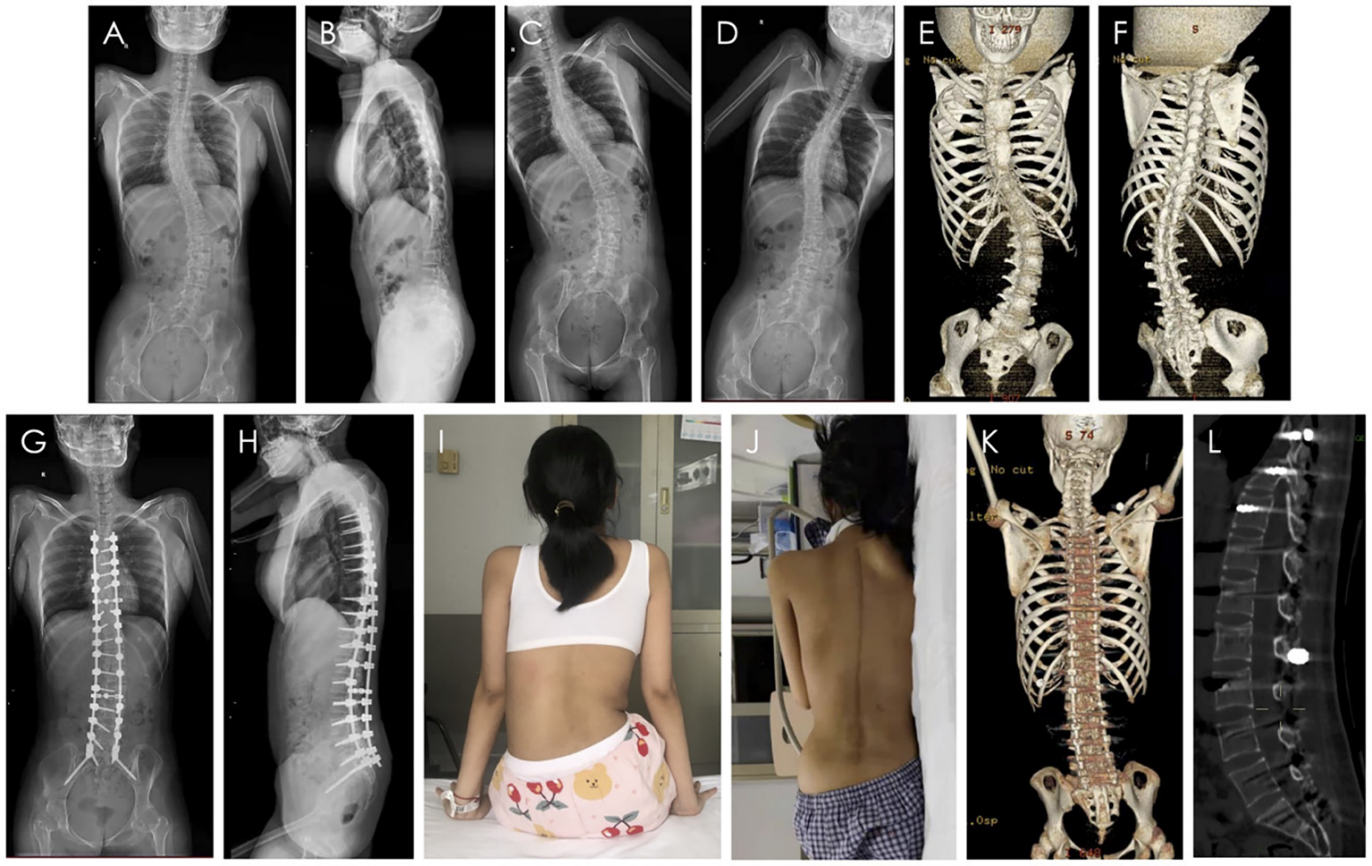
Scoliosis orthopedic surgery. X-ray, CT images and appearance of the SMA patient with scoliosis. **(A)** The anteroposterior view of spinal X-ray before surgery. **(B)** The lateral view of spinal X-ray before surgery. **(C,D)** The left and right bending view of spinal X-ray before surgery. **(E,F)** The three-dimensional CT images before surgery. **(G)** The anteroposterior view of spinal X-ray after surgery. **(H)** The lateral view of spinal X-ray after surgery. **(I)** The appearance of the back before surgery. **(J)** The appearance of the back after surgery. **(K)** The three-dimensional CT images after surgery. **(L)** The sagittal CT images after surgery and the osseous channel are circled in the figure.

Because of the severe pelvic tilt, orthopedic fusion surgery was fixed to the S2 segment, and S2 sacral alar–iliac screws were planted for spinal–pelvic fixation. The surgical procedure was technically successful and well-tolerated. No severe postoperative complications were noted. The scoliosis correction rate was up to 71.4% and her height had increased by 9 cm. Pelvic inclination angle improved from 9.5° (preoperative) to 0.5° (postoperative) and pelvic tilt was well-corrected (Figure 1).

Clinical outcomes of the orthopedic surgery are summarized in Table 1.

**Table 1:** The clinical data and outcome about the orthopedic surgery.

	**Preoperative**	**Intraoperative records**	**Postoperative**
Scoliosis angle (°)	63		18
Height (cm)	140		149
Inclination of pelvis (°)	9.5		0.5
Height difference of shoulders (cm)	2.3		0.5
Height difference of crista iliaca (cm)	1.5		0
Perpendicular distance of S1 and convex point (cm)	3.8		1.9
Weight (kg)	35.3		38
BMI (kg/m^2^)	18.01		17.1
Operation time (min)		437	
Antihepatic time (min)		565	
Operative position		Prone position	
Fusion level		T3-S2 (S2 alar-iliac)	
Intraoperative blood loss (ml)		750	
Heterogenous blood transfusion (ml)		400 (RBC) / 200 (Plasma)	
Autologous blood transfusion (ml)		668	
Number of pedicle screws		30	

### Nusinersen Intrathecal Injection

The intrathecal injection was completed at the 30th day after the orthopedic surgery. In a novel procedure, the osseous channel was left on the vertebral laminae of L3-L4 to facilitate subsequent intrathecal injection in this case. This included partial removal of the superior half of the lamina, the spinous process of L4 and the inferior half of the lamina and the spinous process of L3 with complete excision of the intervening ligamentum flavum.

**Figure 2:**
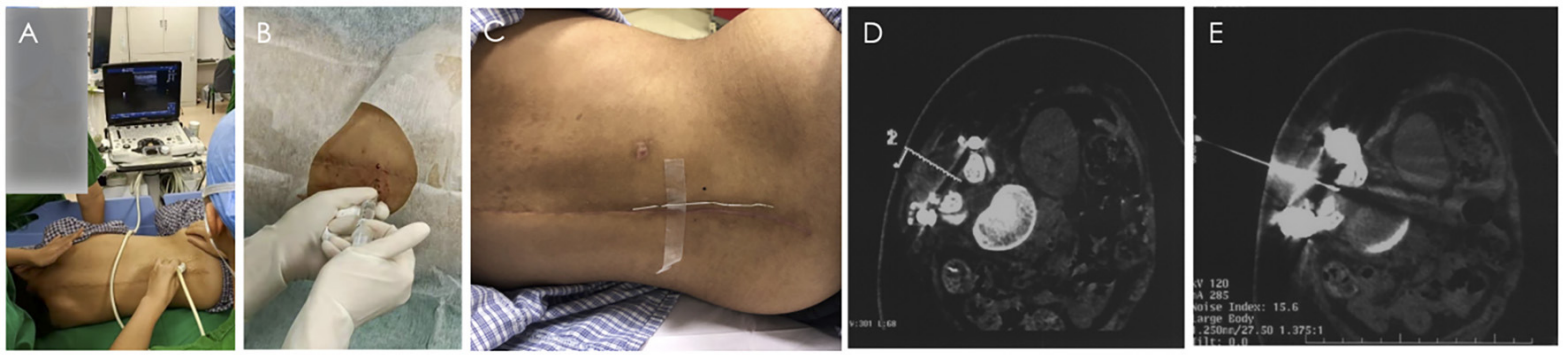
Nusinersen intrathecal injection process under the guidance of B-ultrasound and CT. **(A,B)** Real-time ultrasound-guided lumbar puncture. **(A)** Ultrasound probe position of paramedian sagittal oblique scan at the level of lamina. **(B)** Local anesthesia before intrathecal injection. C-E CT-guided lumbar puncture. **(C)** Place a metal positioning wire at the posterior midline around the lumbar spine. **(D)** Determine the needle insertion route through CT scan, and calculate the needle insertion point on the body surface and needle insertion angle. **(E)** The puncture needle successfully entered the subarachnoid space).

Lumbar punctures were successfully performed with local anesthesia under real-time ultrasound guidance through the laminae channel for the first and second Nusinersen injections (Figures 2A,B), although it was time-consuming to find the anatomical structure in which echo signal was affected by the postoperative local anatomical changes and implants. Considering the comfort and minimization of tissue injury to the patient, lumbar puncture was guided with the help of CT for the third and fourth dosage of Nusinersen (Figures 2C–E). Location, direction, and depth of puncture was quickly and clearly determined by CT, with a much higher resolution. In the CT guidance process, low-dose parameters were set, and the final radiation amount of a single operation was presumably in the very low range of 0.1 (mSv), which was equivalent to the radiation amount of 1 chest CT (0.05–0.24 mSv). Clinical data from the Nusinersen intrathecal injection are shown in Table 2.

**Table 2:** Clinical and motor function evaluation with Nusinersen intrathecal injection.

**Nusinersen intrathecal injection and assessment scale**	**Preoperative**	**1st injection**	**2nd injection**	**3rd injection**	**4th injection**
Guidance mode		Ultrasound-guided	Ultrasound-guided	CT-guided	CT-guided
Operation time (min)		65	49	15	15
Time of puncture (min)		30	40	3	3
VAS score after injection		3	5	1	1
Intraoperative lidocaine dose (ml)		10	10	1	1
MFM32	57	58	58	59	
RULM	34	36	36	36	
HFMSE	33	31	31	31	

### Postoperative Recovery

During the perioperative period, the patient received individualized rehabilitation intervention as soon as possible according to the results of different means of assessment, including: (1) manual active-assistive stretching of lower extremity joints; (2) thoraco-lumbar sacral or those for posture and to promote function; (3) muscle strength training and functional activity exercise; (4) bicycle training with resistance; and (5) respiratory muscle training.

In this study, the MFM 32, RULM, and HFMSE scales were used to evaluate the changes in motor function of the patient (Table 2). After the surgery and three doses of Nusinersen, both MFM32 and RULM scores improved by 2 points, due to improvements in muscle strength and motor function of upper extremities. However, HFMSE scores decreased temporally after surgery, because the surgery damaged the trunk muscles, which affected some activities, such as rolling over. It will probably take some time to recover.

Above all, spinal orthopedic surgery not only increased the thoracic volume of the patient, but also facilitated the rehabilitation exercise process. The motor functions of patients improved significantly after the surgery, four doses of Nusinersen and rehabilitation training.

## Discussion

Spinal muscular atrophy affects motor neurons of the spinal cord and the lower bulbar nuclei (11). It manifests clinically with progressive limb and trunk weakness affecting the proximal trunk muscles more than the distal muscles and the lower limbs more than the upper ones according to the natural history (12). According to the patient's manifestation and gene test result, she was diagnosed SMA, type 3. She received further multidisciplinary treatment in our neuromuscular disorder center.

The spinal deformity develops as a consequence of the generalized weakness and poor ambulatory function (11). Patients with SMA and mild scoliosis who lose ambulatory capacity and became wheelchair-bound show rapid deterioration of their scoliosis and pelvis obliquity (13). Orthopedic surgery is the primary treatment for the patients with neuromuscular scoliosis and pelvic tilt, and is also an important part of the standard care for SMA patients. If the orthopedic corrective surgery is not performed in time, the spinal deformity will significantly be aggravated and the costal arch will gradually impact the iliac crest, resulting in severe lumbar and costal pain. Furthermore, the cardiopulmonary function and gastrointestinal function will deteriorate gradually with the aggravation of chest and abdominal compression, and eventually become life-threatening. The objective of the orthopedic surgery is to correct the tilt and balance of the pelvis, restore the coronal and sagittal balance of the spine, promote potential for upper limb rehabilitation exercise and consequently improve the quality of life of the patient (11). The correction rate for scoliosis depends on the flexibility of the spine. Greater flexibility results in a better correction rate (14). Spinal development usually become mature between the ages of 13–16 accompanied by acceptable curve flexibility, however, spinal flexibility decreases with age (15). Therefore, the optimal age for scoliosis surgery is between 13 and 16 years of age (16). In this case, the balance of the spine and pelvis was improved significantly and the height increased by 9 cm, and the patient was able to sit alone without the assistance of hands after surgery, thus, facilitating the rehabilitation exercises. The trend of improvement of the patient's motor function can be seen in the short term and the further long term follow-up is required.

Nusinersen is an effective, life-changing treatment in children of all ages with SMA (5, 17). It can be administered intrathecally even in children with severe scoliosis or following spine surgery (18, 19). The spinous processes are removed and the vertebra is fixed with the posterior screw rod system during scoliosis correction surgery (20). Due to the lack of anatomical landmarks on the vertebral laminae and deficient flexion of the lumbar spine after orthopedic fusion surgery (21), the space for needle insertion between the adjacent spinous processes is limited. Therefore, the intrathecal injection treatment is very difficult for the patients who have received orthopedic surgery. Cervical puncture can be used for Nusinersen administration. However, this approach was not necessarily due to the high technical success rate of lumbar punctures given that even the slightest error during a cervical puncture operation may cause cervical spinal cord injury, paralysis, and apnea. Transforaminal approach has been used frequently for intrathecal injections after spinal surgery, however, there is a risk of injuries to nerve roots and vessels, which pass through the foramen. In order to preserve the channel for intrathecal injection, an L3-L4 laminectomy can be performed at the same time as spinal correction and fusion surgery in this patient.

The first two lumbar punctures were performed with local anesthesia under real-time ultrasound guidance while the third and fourth ones were administered with CT guidance through the laminae channel.

Compared with the CT guidance, ultrasound guidance is available readily at the bedside and radiation-free, but it is important for a clinically experienced physician to perform the procedure because metal artifacts such as implants can interfere with the ultrasound. It always takes a period of time to find the vertebral space using ultrasound, and the longer operation time brings more discomfort to the patient (22). CT-guided intrathecal injection is a feasible method of Nusinersen administration for patients with difficult lumbar puncture because of the severe scoliosis or spinal stabilization with a high technical success rate (23). In this case, CT-guided puncture required less operative time, a lower local anesthetic dose, and lower VAS score after the procedure compared with ultrasound guidance. Considering the advantages and disadvantages of both guidance approaches, we recommend real-time ultrasound guidance for the first intrathecal injection of Nusinersen for SMA patients after spinal surgery, which can be replaced by CT guidance if the first puncture process is difficult.

In summary, scoliosis orthopedic surgery combined with Nusinersen intrathecal injection is an effective and efficient procedure for SMA patients with scoliosis. The patient benefited from the osseous channel left during orthopedic surgery. Not only were the scoliosis and pelvic tilt significantly corrected, but the intrathecal injection was performed safely. As with all the single case studies, further studies with larger sample sizes are needed.

## Data Availability Statement

The original contributions presented in the study are included in the article/supplementary material, further inquiries can be directed to the corresponding authors.

## Ethics Statement

The studies involving human participants were reviewed and approved by the Ethics Committee of Peking University First Hospital (No. 2015[916], Beijing, China). Written informed consent to participate in this study was provided by the participants' legal guardian/next of kin. Written informed consent was obtained from the individual(s), and minor(s)' legal guardian/next of kin, for the publication of any potentially identifiable images or data included in this article.

## Author Contributions

BX was a major contributor in writing the manuscript and completed the orthopedic surgery. CW designed the study, performed the lumbar punctures, and revised the manuscript. BX and CW both contributed equally to this work. XH facilitated the lumbar punctures. WL, ZH, and CQ all revised the manuscript. CL revised the manuscript, supervised the lumbar punctures, and completed the orthopedic surgery. HX designed the study and also revised the manuscript. All these authors critically revised and approved the final manuscript.

## Funding

This work was supported by the grants from the National Natural Science Foundation of China (No. 82171393); the National Key Research and Development Program of China (No. 2016YFC0901505), and Beijing Key Laboratory of Molecular Diagnosis and Study on Pediatric Genetic Diseases (No. BZ0317).

## Conflict of Interest

The authors declare that the research was conducted in the absence of any commercial or financial relationships that could be construed as a potential conflict of interest.

## Publisher's Note

All claims expressed in this article are solely those of the authors and do not necessarily represent those of their affiliated organizations, or those of the publisher, the editors and the reviewers. Any product that may be evaluated in this article, or claim that may be made by its manufacturer, is not guaranteed or endorsed by the publisher.
